# Additional Primary Tumors Detected Incidentally on FDG PET/CT at Staging in Patients with First Diagnosis of NSCLC: Frequency, Impact on Patient Management and Survival

**DOI:** 10.3390/cancers15051521

**Published:** 2023-02-28

**Authors:** Ken Kudura, Nando Ritz, Arnoud J. Templeton, Marc Kissling, Tim Kutzker, Robert Foerster, Martin H. K. Hoffmann, Kwadwo Antwi, Michael C. Kreissl

**Affiliations:** 1 Department of Nuclear Medicine, Sankt Clara Hospital, 4058 Basel, Switzerland; 2 Department of Radiology, Sankt Clara Hospital, 4058 Basel, Switzerland; 3 Faculty of Medicine, University of Basel, 4001 Basel, Switzerland; 4 Sankt Clara Research, 4002 Basel, Switzerland; 5 Faculty of Applied Statistics, Humboldt University, 10117 Berlin, Germany; 6 Department of Radiooncology, Cantonal Hospital Winterthur, 8400 Winterthur, Switzerland; 7 Division of Nuclear Medicine, Department of Radiology and Nuclear Medicine, University Hospital Magdeburg, 39120 Magdeburg, Germany

**Keywords:** positron emission tomography computed tomography, PET/CT, FDG-PET/CT, lung cancer, NSCLC, staging, multiple primary malignancies

## Abstract

**Simple Summary:**

The improved survival rates of lung cancer patients have led to an increased number of patients experiencing multiple primary malignancies during the course of their life. Therefore, a tool that can evaluate the risk of multiple primary cancers and detect them as early as possible could be of great clinical relevance. [^18^F]fluoro-D-glucose positron emission tomography/computed tomography (FDG-PET/CT) has been widely used and recommended as standard care for clinical staging in lung cancer by international guidelines. However, only very limited data on the detection of additional primary malignancies on FDG-PET/CT at initial staging in non-small cell lung cancer (NSCLC) patients have been published in recent years. Therefore, we aimed to assess the frequency of additional primary malignancies detected incidentally on FDG-PET/CT at staging in patients with the initial diagnosis of NSCLC. Moreover, their impact on patient management and survival was assessed. FDG-PET/CT performed for staging might be a valuable tool to identify additional primary tumors in NSCLC patients. In our study, FDG-PET/CT identified a significant number of additional primary malignancies. Upon validation, additional primary tumors might have substantial implications for patient management clinicians that they should be aware of. Early detection of additional primary tumors together with interdisciplinary patient management may lead to similar survival rates as compared to patients with NSCLC only.

**Abstract:**

We aimed to assess the frequency of additional primary malignancies detected incidentally on [^18^F]fluoro-D-glucose positron emission tomography/computed tomography (FDG-PET/CT) at staging in NSCLC patients. Moreover, their impact on patient management and survival was assessed. Consecutive NSCLC patients with available staging FDG-PET/CT between 2020 and 2021 were retrospectively enrolled. We reported whether further investigations of suspicious findings presumably not related to NSCLC were recommended and performed after FDG-PET/CT. Any additional imaging, surgery or multimodal management was considered as an impact on patient management. Patient survival was defined using overall survival OS and progression-free survival PFS. A total of 125 NSCLC patients were included, while 26 findings in 26 different patients were suspicious for an additional malignancy on FDG-PET/CT at staging. The most frequent anatomical site was the colon. A total of 54.2% of all additional suspicious lesions turned out to be malignant. Almost every malignant finding had an impact on patient management. No significant differences were found between NSCLC patients with suspicious findings versus no suspicious findings with regards to their survival. FDG-PET/CT performed for staging might be a valuable tool to identify additional primary tumors in NSCLC patients. Identification of additional primary tumors might have substantial implications for patient management. An early detection together with interdisciplinary patient management could prevent a worsening of survival compared to patients with NSCLC only.

## 1. Introduction

Cancer is the leading cause of death worldwide, with almost 10 million cancer deaths in 2020 and with an estimated 47% rise in the global cancer burden from 2020 to 2040 [1,2]. The rapid rise of cancer as the leading cause of death globally reflects converging trends such as declining mortality rates of cardiovascular diseases in the aging population in many countries but also social and economic development in several regions of the world, particularly in transitioning countries [1,3,4].

Lung cancer is the leading cause of cancer death and second most commonly diagnosed cancer worldwide, with two times higher mortality and incidence rates in men than in women. Despite heterogeneous geographic trends reflecting different smoking habits, most countries are still observing a rising incidence of lung cancer [1], with non-small lung cancer (NSCLC) accounting for 85% of the cases [5,6]. Significant improvement in early diagnostic tools and treatment modalities of lung cancer, together with a growing aged population, led to a booming of cancer survivors [1,2,7,8,9,10]. The improved survival rates of lung cancer patients increased the number of patients experiencing multiple primary malignancies in the course of their life [4,11,12,13,14,15,16,17,18,19,20,21,22,23]. Therefore, a tool which is able to evaluate the risk of multiple primary cancers and detect them as early as possible could be of great clinical relevance.

2-deoxy-2-[^18^F]fluoro-D-glucose positron emission tomography/computed tomography (FDG-PET/CT) has been validated as an accurate diagnostic tool for detecting asymptomatic recurrence of NSCLC during postoperative follow-up or additional primary tumors in NSCLC patients after curative treatment [24,25,26]. However, only very limited data on the detection of additional primary malignancies on FDG-PET/CT at initial staging in NSCLC patients have been published in recent years [27], even though FDG-PET/CT has been widely used and recommended as standard care for clinical staging in lung cancer by international guidelines [28,29]. Tumor cells overexpress numerous glucose transporter proteins promoting the uptake of glucose into tumor cells. The principle of FDG-PET/CT is the intravenous injection of FDG, which, acting as glucose analog, will be taken up intracellularly by glucose transporter. Once in the tumor cells, FDG will be phosphorylated, so it cannot be further metabolized. Upon phosphorylation, FDG will be accumulated in the tumor cells over time and so be visualized on imaging. Thus, hybrid imaging is a non-invasive approach to provide biological information (on the metabolism, for instance), but also morphological features (such as size, shape) of a tumor in the same modality using PET/CT or PET/MR, whenever a better anatomical mapping or superior soft tissue contrast is required [23].

Therefore, we aimed to assess the frequency of additional primary malignancies detected incidentally on FDG-PET/CT at staging in patients with initial diagnosis of NSCLC, as much as their impact on patient management and survival.

## 2. Materials and Methods

### 2.1. Patient Cohort

Consecutive patients with pathologically confirmed NSCLC treated at the Sankt Clara Hospital in Basel (Switzerland) and available NSCLC staging FDG-PET/CT scan performed at our institution before any treatment between 1 January 2020 and 31 December 2021 were retrospectively reviewed. The cohort was then dichotomized into two groups: initial diagnosis of NSCLC with further finding suspicious of additional malignancy versus no further suspicious findings on baseline FDG-PET/CT.

### 2.2. Baseline Characteristics

Patient age, sex, body mass index BMI, histopathological subtype of NSCLC and clinical stage (8th edition of the American Joint Committee on Cancer AJCC stage) were used as baseline characteristics.

### 2.3. FDG-PET/CT Acquisition

FDG-PET/CT was performed in clinical routine for NSCLC staging in accordance with our department standard protocol (e.g., from the vertex of the skull to the thighs in supine position with iodinated contrast medium in absence of renal impairment or allergy). All FDG-PET/CT scans considered for the purpose of this single-center retrospective study were carried out on a discovery PET/64-detector CT scanner (GE Discovery Molecular Insights—DMI PET/CT, GE Healthcare, Waukesha, WI, USA) at our institution.

### 2.4. FDG-PET/CT Report Review

We retrospectively reviewed manually all FDG-PET/CT reports searching for lesions reported in clinical routine as suspicious and presumably not related to the primary tumor (NSCLC) by two reporting physicians (both boards certified in radiology and nuclear medicine, respectively, 6 and over 10 years of experience in hybrid imaging). Lesions were reported as suspicious whenever the uptake was higher than the physiological uptake of the surrounding tissue and clearly measurable. Evidence or absence of morphological correlate on diagnostic CT scan (mostly with iodinated contrast medium) was also used by the reporting physicians for interpretation.

Additionally, we reported whether further investigations of the suspicious lesion were recommended by the reporting physicians and performed after FDG-PET/CT. The nature of the lesion was reported as suspicious, just as the impact on patient management and survival were also assessed.

- Number of lesions suspicious of an additional malignancy on FDG-PET/CT at staging

The number of suspicious lesions was documented per patient and anatomical site.

b. Further investigations recommended after FDG-PET/CT

Any recommendation of further imaging, such as CT or magnetic resonance imaging MRI, as well as imaging-guided biopsy of the suspicious findings by PET/CT reporting physicians were systematically documented. Subsequently, we recorded whether the recommended modality was performed following the FDG-PET/CT scan.

c. Nature of suspicious lesions

The nature of all considered suspicious lesions was determined in clinical routine using radiological properties (such as evidence/absence of contrast medium enhancement or increase in size on follow-up imaging shortly after FDG-PET/CT) or histopathological findings after imaging-guided biopsies.

d. Impact on patient management

Any additional imaging, surgery or multimodal management (e.g., combination of imaging, surgery or radiotherapy) of the suspicious finding performed after FDG-PET/CT and reported in clinical records including interdisciplinary tumor board decisions were retrospectively considered as having an impact on patient management.

e. Impact on patient survival

Overall survival OS (date of NSCLC diagnosis to death or last follow-up) and progression-free survival PFS (date of NSCLC diagnosis to disease death or progression based on imaging and/or clinical findings) were reported for the dichotomized cohort. Both endpoints for patient survival were assessed on 19 August 2022.

### 2.5. Statistical Analysis

All statistical computations were performed using R (version 4.1.1). Categorical variables were described using frequencies. Continuous variables were characterized with mean and standard deviation SD. A log rank test was performed to compare survival rates within the dichotomized cohort. Statistical significance was accepted at *p* < 0.05.

## 3. Results

### 3.1. Baseline Characteristics

One hundred and twenty-five patients with initial diagnosis of NSCLC fulfilled all above-listed inclusion criteria. Afterwards, inclusion patients were dichotomized into two groups: NSCLC patients with findings suspicious for an additional malignancy (*n* = 26) versus no suspicious findings (*n* = 99) on baseline FDG-PET/CT.

Significant differences with regard to sex distribution within the two groups were observed (*p* = 0.04). In fact, almost 70% of the population with findings suspicious for an additional malignancy were male patients, while female patients dominated the cohort with no suspicious finding (53.5%).

However, no further significant differences with regard to baseline characteristics were found among the dichotomized cohort as presented in Table 1.

### 3.2. Anatomical Site of Suspicious Findings

**Figure 1:**
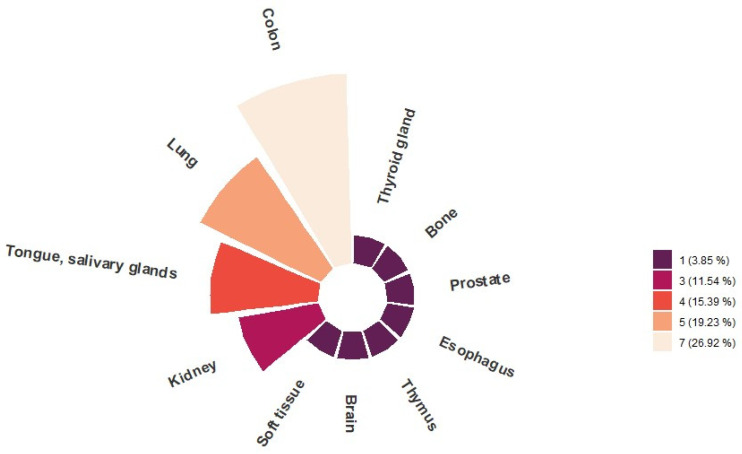
Anatomical distribution of all findings suspicious of additional malignancy (*n* = 26) detected on FDG-PET/CT performed for staging in patients with initial diagnosis of NSCLC (*n* = 125).

**Figure 2:**
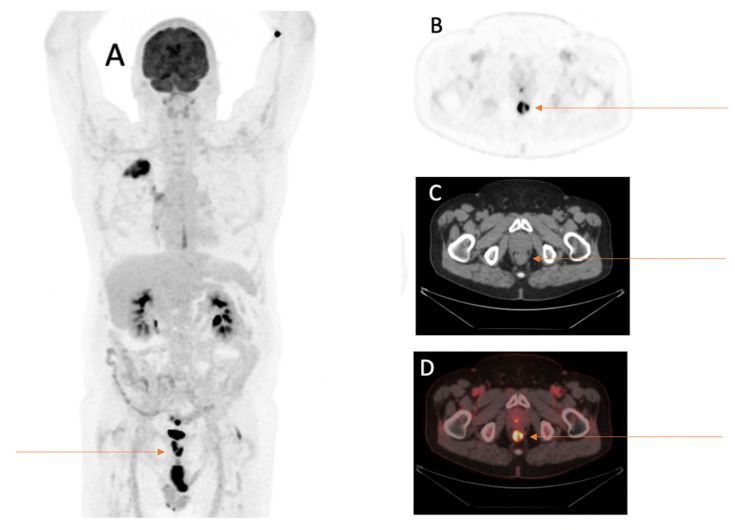
Sixty-nine-year-old male patient with first diagnosis of NSCLC (TTF1 positive adenocarcinoma of the right lower lobe pT2a, pN0, M0) underwent FDG-PET/CT for staging. FDG-PET/CT scan displayed an incidental FDG-avid wall thickening of the rectum. (**A**) MIP image after the intravenous injection of 245 MBq ^18^F-FDG. (**B**) PET image of the rectum. (**C**) CT image of the rectum. (**D**) Fused PET/CT image of the rectum. After further investigations following the FDG-PET/CT, the wall thickening was histopathologically confirmed as microsatellite stable adenocarcinoma of the rectum T3 cN2 cM0, 5 cm oral to the anus.

**Figure 3:**
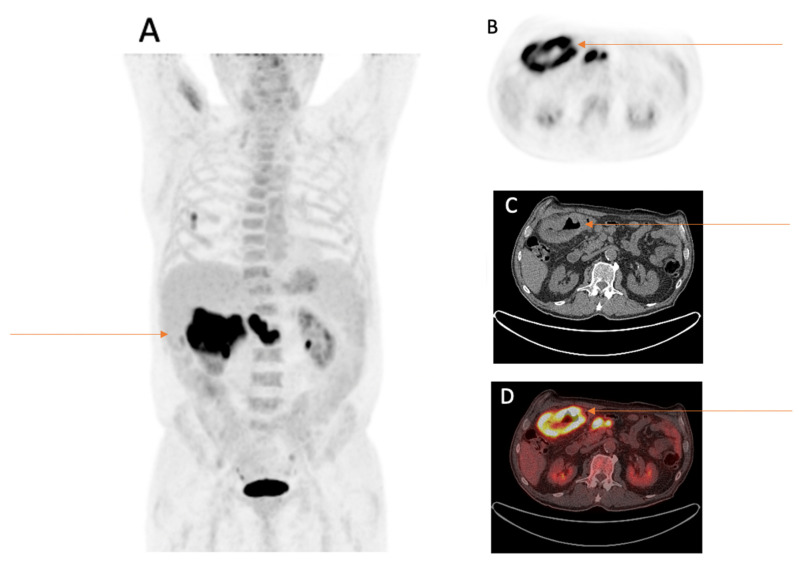
Eighty-three-year-old male patient with first diagnosis of NSCLC (TTF1 positive adenocarcinoma of the right lower lobe pT1b, pN0, M0) underwent FDG-PET/CT for staging. FDG-PET/CT scan displayed an incidental FDG-avid wall thickening of the right colon flexure with three FDG-avid locoregional lymph nodes. (**A**) MIP image after the intravenous injection of 322 MBq ^18^F-FDG. (**B**) PET image of the right colon flexure. (**C**) CT image of the right colon flexure. (**D**) Fused PET/CT image of the right colon flexure. After further investigations, the wall thickening was histopathologically confirmed as microsatellite stable adenocarcinoma of the colon ascendens pT3 N2a (4/26), V1, L1, R0.

Twenty-six findings in twenty-six different patients were rated by reporting physicians as suspicious of additional malignancy. The most frequent anatomical sites for suspicious findings were the colon with 26.9% (*n* = 7), followed by the lung with 19.2% (*n* = 5), the tongue/salivary gland with 15.4% (*n* = 4) and the kidneys with 11.5% (*n* = 3). In addition, about a quarter (27.0%) of the suspicious findings were equally distributed between the following anatomical sites: the thyroid gland with 3.9% (*n* = 1), bone with 3.9% (*n* = 1), prostate with 3.9% (*n* = 1), esophagus with 3.9% (*n* = 1), thymus with 3.9% (*n* = 1), brain with 3.9% (*n* = 1) and soft tissue with 3.9% (*n* = 1), as presented in Figure 1, Figure 2 and Figure 3.

### 3.3. Recommended Investigations and Nature of Suspicious Findings

Further investigations of suspicious findings were often recommended by the reporting physicians (65.4%, 17 out of 26 cases). Radiological short-term follow-up was overall most frequently recommended for further investigations (58.8%, 10 out 17 cases) with CT as the most requested modality. However, imaging-guided biopsy was the preferred modality to investigate suspicious lesions of the colon (80.0%, 4 out of 5 cases), as much as single suspicious lesions located in esophagus, tongue/salivary gland and thymus. Interestingly, almost all pulmonary (80.0%, 4 out of 5 cases) and all renal (100.0%, 3 out of 3 cases) findings reported as suspicious for additional malignancy were also considered eligible for further radiological investigations by the reporting physicians. Two NSCLC patients died shortly (a few days) after FDG-PET/CT scan was performed for staging, so that the recommended investigations into the suspicious findings could not be performed. Otherwise, in 94.4% of the cases (17 out of 18 cases), recommendations for further investigations of suspicious findings by reporting physicians were performed shortly after FDG-PET/CT.

**Figure 4:**
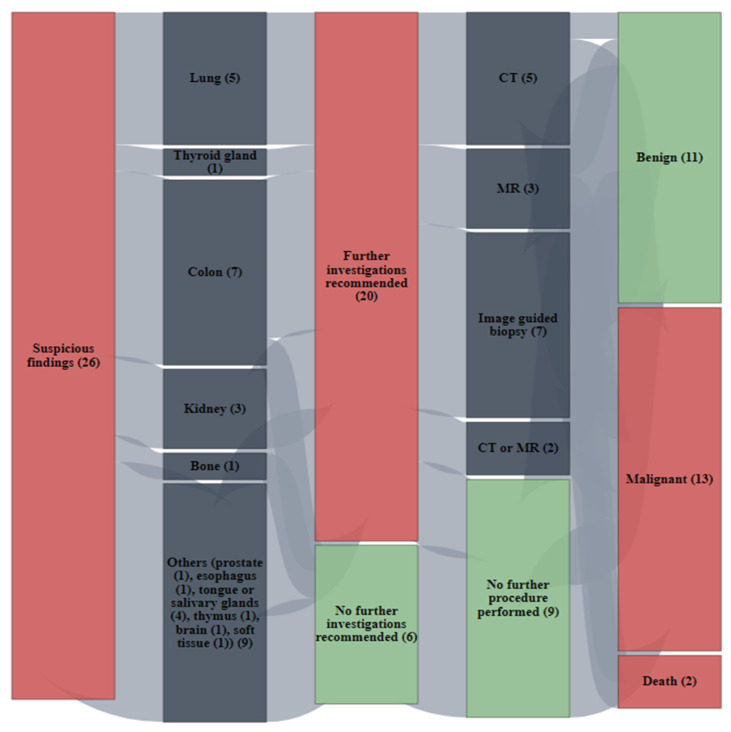
Sankey plots display the number of suspicious findings (total and per organ) detected incidentally on FDG-PET/CT in NSCLC patients at staging. It is also displayed, whether further investigations into the suspicious findings were recommended or not by the reporting physicians, as much as the nature of the findings after examinations.

The nature of 24 out of 26 suspicious lesions could be clarified; two patients passed away shortly after staging before any investigation. Every second additional lesion (54.2%, 13 out of 24 lesions) that was reported as suspicious on FDG-PET/CT and further examined following FDG-PET/CT in clinical routine turned out to be malignant, as presented in Figure 4.

### 3.4. Further Description of Suspicious Findings

**Figure 5:**
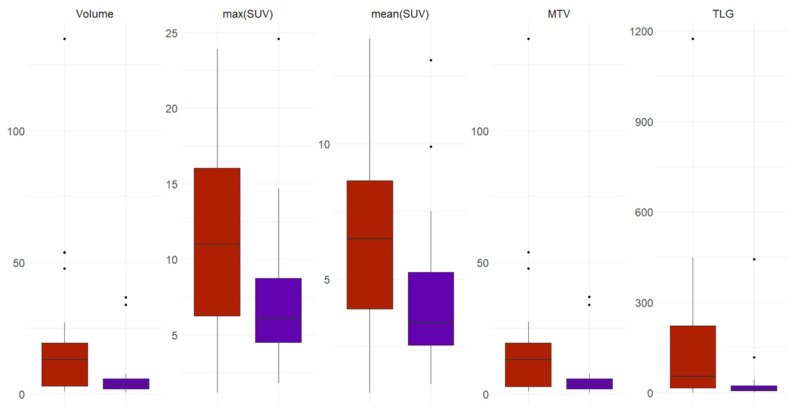
Box plots display the differences in volume and metabolic parameters (SUVmax, SUVmean, MTV and TLG) between the primary tumors (in red) and suspicious findings (purple).

Volumes (in ml) and metabolic parameters (e.g., SUV max, mean, MTV and TLG) of all suspicious findings were lower than those of the primary tumors, as displayed in Figure 5.

The following malignancies unrelated to the primary tumor NSCLC (*n* = 13) could be confirmed after investigation of the suspicious findings: prostate cancer in one patient, renal cell cancer in one patient, multiple myeloma in one patient, tongue base cancer in one patient, esophageal cancer in one patient, concomitant second lung cancer in three patients and colorectal cancer in five patients.

### 3.5. Impact on Patient Management

**Figure 6:**
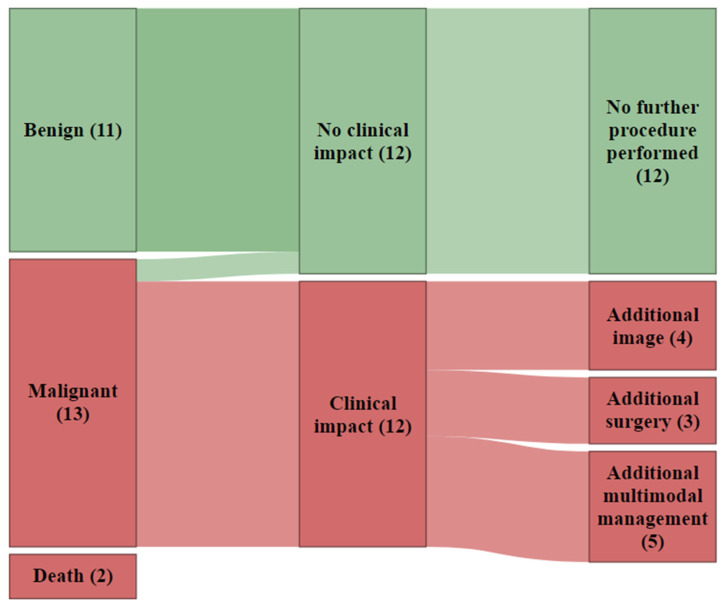
Sankey plots displays, whether patient management was influenced by findings reported as suspicious of additional malignancy on FDG-PET/CT at staging in NSCLC patients with regards to their nature after further examinations following FDG-PET/CT.

Almost every single finding suspicious (92.3%) on staging FDG-PET/CT and found to be malignant after further evaluation had an impact on patient management. Mostly additional multimodal management (e.g., combination of imaging with surgery or radiotherapy) was required (41.7%), followed by additional imaging (33.3%) or surgery (25.0%). Figure 6.

### 3.6. Impact on Patient Survival

No significant differences were found between patients with suspicious findings versus patients with no suspicious findings with regard to their long-term survival.

Table 2 shows the long-term survival of NSCLC patients with incidental findings suspicious of additional malignancy versus no suspicious findings at baseline using overall survival and progression-free survival as endpoints for survival.

**Figure 7:**
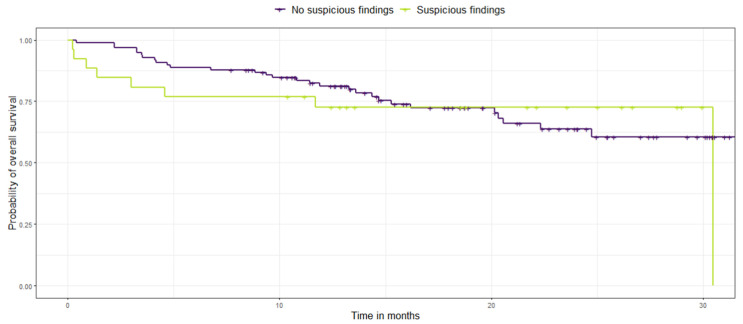
Kaplan-Meier survival curves display the probability of overall survival (in months) in NSCLC patients with additional suspicious findings (lemon green) versus no suspicious findings (purple).

**Figure 8:**
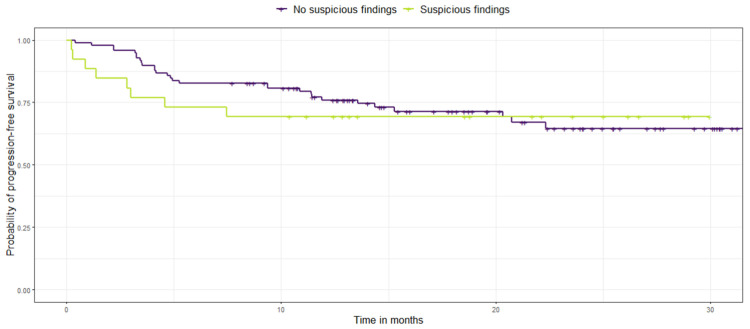
Kaplan-Meier survival curves display the probability of progression-free survival (in months) in NSCLC patients with additional suspicious findings (lemon green) versus no suspicious findings (purple).

Furthermore, a log rank test was performed to compare survival rates within the dichotomized cohort. In fact, no significant difference was found in their probability of overall survival (*p* = 0.92), nor their probability of progression-free survival (*p* = 0.75) Figure 7 and Figure 8.

## 4. Discussion

We aimed to assess the frequency of additional primary malignancies detected incidentally on staging FDG-PET/CT in patients with the initial diagnosis of NSCLC, as much as their impact on patient management and survival.

Our investigations brought interesting results to light, which might be clinically relevant in the near future since the number of patients experiencing multiple primary malignancies is globally expected to rise rapidly and considerably in the coming decades [4,11,12,13,14,15,16,17,18,19,20,21,22,23].

A total of 125 consecutive patients with the initial diagnosis of NSCLC fulfilled our inclusion criteria. In every fifth NSCLC patient, a lesion suspicious for an additional malignancy was reported on staging FDG-PET/CT, a vast majority of these patients being older men.

Interestingly, colon followed by lung and ORL organs such as tongue and salivary glands were the most frequent anatomical sites for suspicious lesions not related to NSCLC at staging. Further investigations of the suspicious findings were often recommended by reporting physicians with CT as the most requested modality. However, imaging-guided biopsy was the preferred modality to investigate suspicious lesions located in the colon, esophagus, tongue/salivary gland and thymus. In our institution, almost all recommendations for further investigations of suspicious findings by the reporting physicians were performed shortly after FDG-PET/CT in clinical routine. A total of 54.2% of the lesions were reported as suspicious on FDG-PET/CT and further examined following FDG-PET/CT turned out to be malignant. Almost every malignant finding had an impact on patient management, such as additional multimodal management, followed by additional imaging or surgery.

Finally, no significant differences were found among the dichotomized cohort concerning their long-term survival.

In light of these innovative insights, the following four major key points should be highlighted.

First of all, additional primary malignancies were frequent. In fact, in one out of ten patients with the initial diagnosis of NSCLC, an additional primary malignancy incidentally detected on FDG-PET/CT at staging could be validated. The incidence of multiple primary malignancies has increased and continued to grow in recent decades [23]. According to Yilmaz et al., the occurrence of multiple malignancies may be explained by several factors, such as alcohol and smoking, environmental carcinogens, genetic factors and hormones. In fact, smokers might have a significantly higher risk for multiple primary malignancies [7].

A particular caution applies to a few organs, including colon, lung and ORL organs such as tongue and salivary glands given their higher rates of additional primary malignancies, especially in older men with NSCLC. Our results are consistent with recently published investigations. Chou W-R et al. reported that most common concomitant malignancies in men with lung cancer were lung, colon, gastric and prostate cancers [18]. Liu et al. also observed higher incidence of associated cancers, such as lung cancer with upper aerodigestive tract cancer [30].

Furthermore, additional primary malignancies were clinically relevant since every additional primary malignancy validated in NSCLC patients shortly after baseline FDG-PET/CT had an impact on patient management, mostly requiring further interdisciplinary patient care. Consequently, the detection of additional primary malignancies might have significant implications for patient health needs and costs that clinicians should be aware of.

Finally, the arising of two concomitant primary malignancies in NSCLC patients did not lead to a worse survival compared to patients with NSCLC only. Since no difference in survival was found between these patients, the question of whether further investigations of suspicious findings was necessary after FDG-PET/CT considering the potential additional stress should also be addressed. Our data suggested that a vast majority of additional malignancies often detected at an early stage led not only to additional imaging but often to additional surgery or radiotherapy. Thus, the early detection of additional primary malignancies at staging in conjunction with an early interdisciplinary patient management could have played a decisive role in the comparable survival of these patients to patients with NSCLC only.

In knowledge of the limited literature published in the past 10 years, we came across only one comparable work, published 10 years ago. Lin M. et al. reviewed 649 FDG-PET/CT scans performed between February 2006 and July 2010 searching for unexpected premalignancy or additional malignancy at staging in NSCLC patients. Their results published in 2012 were consistent with our results. A total of 12% of the patients were identified with premalignancy or additional malignancy. Moreover, whenever validated, additional primary malignancies had a significant impact on patient management [27].

However, three main limitations of our investigations should also be addressed.

This single-center retrospective study was conducted in Switzerland, which might have led to geographic particularities such as radon exposure, alcohol and tobacco consumption, as much as a good access to health facilities with interdisciplinary patient care.

Moreover, given the chosen retrospective study design, no further information about patient personal behavior (alcohol and tobacco consumption), occupational exposure, socioeconomic situation and lifestyle could be retrospectively obtained during our investigations. This information might have played an important role in better understanding the relationship between NSCLC and the detected concomitant additional primary malignancies. Finally, the size of our cohort considering two consecutive years for patient enrollment might be a further limitation of our data. Further larger (for instance, 5 consecutive years) prospective, multicentric studies with special care to geographic disparities and personal risk for multiple malignancies might be needed to overcome these limitations and confirm our data.

## 5. Conclusions

FDG-PET/CT performed for staging might be a valuable tool for identifying additional primary tumors in NSCLC patients as it identified a significant number of additional primary malignancies. Identification of additional primary tumors might have substantial implications for patient management. An early detection of additional primary tumors together with interdisciplinary patient management could prevent a worsening of survival compared to patients with NSCLC only.

## Figures and Tables

**Table 1:** Baseline characteristics of all included patients with first diagnosis of NSCLC (*n* = 125) with findings suspicious of additional malignancy (*n* = 26) and without suspicious findings (*n* = 99).

Characteristics	No Suspicious Finding (*n* = 99)	Suspicious Finding (*n* = 26)	*p*-Value
**Age** Mean ± SD (years)	71.5 ± 9.1	74.3 ± 11.0	0.23
**Sex** Male Female	46.5% 53.5%	69.2% 30.8%	0.04
**BMI** Mean ± SD (kg/m^2^)	26.23 ± 6.1	25.88 ± 5.4	0.77
**Histopathological subtype** Adenocarcinoma Squamous cell carcinoma Large cell carcinoma Neuroendocrine Tumor Not specific	47.5% 15.2% 12.1% 22.2% 3.0%	46.1% 7.7% 11.6% 34.6% 0.0%	0.68
**Clinical staging** I II III IV	22.2% 6.1% 36.4% 35.3%	26.9% 3.8% 46.2% 23.1%	0.64

**Table 2:** Long-Term Survival of NSCLC patients with incidental finding suspicious of additional malignancy versus no suspicious finding at baseline using overall survival and progression-free survival as endpoints for survival.

Endpoints for Long-Term Survival	No Suspicious Finding (*n* = 99)	Suspicious Finding (*n* = 26)	*p*-Value
**Overall survival** Mean ± SD in months	16.6 ± 8.2	16.4 ± 10.4	0.92
**Progression-free survival** Mean ± SD in months	15.9 ± 8.7	15.1 ± 10.4	0.75

## Data Availability

The data presented in this study are available on request from the corresponding author.

## Abbreviations

NSCLC	Non-small lung cancer
FDG-PET/CT	2-deoxy-2-[^18^F]fluoro-D-glucose positron emission tomogra phy/computed tomography
EKNZ	Ethikkommission Nordwest- und Zentralschweiz
BMI	Body mass index
AJCC	American Joint Committee on Cancer (8th edition)
MRI	Magnetic resonance imaging
OS	Overall survival
PFS	Progression-free survival
SD	Standard deviation
MIP	Maximum intensity projection
